# A Gyroscope Bias Estimation Algorithm Based on Map Specific Information

**DOI:** 10.3390/s18082534

**Published:** 2018-08-02

**Authors:** Tian Tan, Ao Peng, Junjun Huang, Lingxiang Zheng, Gang Ou

**Affiliations:** School of Information Science and Engineering, Xiamen University, Xiamen 361000, China; tantian@stu.xmu.edu.cn (T.T.); 23320161153420@stu.xmu.edu.cn (J.H.); lxzheng@xmu.edu.cn (L.Z.); ougang@xmu.edu.cn (G.O.)

**Keywords:** gyro bias, quaternion, bias estimation

## Abstract

In an inertial navigation system, especially in a pedestrian dead-reckoning system, gyroscope bias can demonstrably reduce positioning accuracy. A novel gyroscope bias estimation algorithm is proposed, which estimates the bias of a gyroscope under any set of angle observations. Moreover, a method for obtaining Euler angles using map corridor information is proposed. The heading information obtained from a map is used to estimate the bias, and the estimated bias is used to correct the trajectories. Experimental results show that it is feasible for the algorithm to estimate the bias of the gyroscope.

## 1. Introduction

Accurate attitude information (pitch, roll, and heading angles) is an essential part of good navigation results. In various systems that rely on inertial sensors for navigation and positioning, attitude information is obtained mainly using a gyroscope. However, because of factors such as materials and manufacturing processes, the values measured by gyroscopes include errors. Especially for low-cost gyroscopes, large random errors in measurement occur [1]. Also, in long-term navigation, errors seriously impede estimation accuracy and even result in positioning being unavailable. Therefore, it is necessary to estimate and eliminate the bias of the gyroscope.

Some researchers have estimated gyroscope bias based on quaternion. Vik et al. [2] proposed a scheme to express nonlinear kinematics using quaternions. It is assumed that the bias model of the gyroscope exponentially decays, but that does not correspond to the actual situation. Boskovic et al. [3,4], proposed a quaternion-based nonlinear deviation estimator and coupled the estimator with an adaptive sliding control strategy. Because the object in these papers is a spacecraft, the motion model is more constrained. The estimator assumes that the change of attitude of the spacecraft will not exceed 180 degrees and the change cannot be applied to some situations with large changes in attitude. The stability between coupled observers, controllers, and spacecraft dynamics is not formally established. Yuan [5] proposed a 16-stage rotation scheme for a dual-axis rotary inertial navigation system (INS) that can compensate for gyro drift errors without introducing additional system error accumulation. Wu et al. [6] proposed a Rotary Inertial Measurement Unit (RIMU) method. The accelerometer is used to measure the relationship between the components of the gravity acceleration in each axis and the angular velocity of the gyroscope. Using the dial to get relationship under different coordinate axes and to obtain the multiple equations which can be used to estimate bias. Although these methods can estimate the deviation of the gyroscope, they require a turning tool that can turn any angle around any axis. The bias of the gyroscope is obtained from experiments, but the gyro bias cannot be tracked in real time, and corrections cannot be made. Applications are limited. For example, it is difficult to apply these methods to the indoor positioning of a pedestrian. Thienel et al. [7] tracked the bias of the gyroscope in real time as the bias converged exponentially. However, this method is applied in the case of aerospace space. The motion model, which is not applicable to robots or walking navigation, has limitations. Benallegue et al. [8], used an inertial vector measurement to design an adaptive attitude tracker for a rigid body system. The angular velocity and attitude asymptotically converge to their expected values. However, their method requires controlled inputs and there is no feedback of the attitude. In addition, to estimate the gyro bias, some methods must be combined with magnetometers [9], GPS [10,11], or other external aids. However, these methods are prone to errors when unpredictable distortion of the Earth’s geomagnetic field occurs [12,13], GPS signals are not available [14], or when external auxiliary sensors cannot be installed, e.g., in an urban environment and in a building with many steel structures [15].

We propose a method that does not rely on specific sensor information. By solving the attitude angle matrix differential equation of the rigid body rotation, the recurrence relation between the quaternion of the real angle and the sensor is obtained. Therefore, when there is an arbitrary set of observations of the Euler angle input, the deviation of the gyroscope is tracked and corrected in real time. We use the long corridor information of a map to obtain true heading information. In Section 2, the method and formula derivation are introduced, and an angle observation method is provided. In Section 3, the Euler angles obtained from this angle observation method are combined with the gyro bias estimation algorithm to obtain simulation results. The results show that it is feasible for the algorithm to estimate the bias of the gyroscope.

## 2. Algorithm Introduction

### 2.1. Method

After the output of the gyroscope is processed, the angle (also called Euler angle) of the attitude of the current carrier in the geographic coordinate system is obtained and expressed as follows:

(1) $$u_{k}=f(\Omega_{k})$$

where, $\Omega_{k}$ represents the output of the gyroscope at the current moment; $u_{k}$ represents the attitude angle of the object at the current moment. For Equation (1), a good attitude is obtained with a more accurate gyro output. Good positioning results and navigation trajectory are obtained with an accurate attitude angle. The output of the gyroscope inevitably contains the deviation of the gyroscope, which affects the Euler angle calculations. Over time, the errors accumulate and eventually cause the output information from the gyroscope to be completely unreliable. So the problem of how to acquire accurate gyroscope output must be solved. Likewise, accurate observations of the angle can be obtained through maps, magnetic fields or other methods. By using the long corridor information of the map, we can obtain the true heading information and inversely calculate the more accurate three-axis rotation speed:

(2) $$\Omega_{k}=f^{-1}(u_{k}{}^{r})$$

$\Omega_{k}$ is compared with the output of the gyroscope to obtain the gyro bias. By compensating the gyroscope output with the obtained gyro bias, accurate gyro output is obtained.

### 2.2. Formula Derivation

The nature of the quaternion is very favorable for expressing rotational information [16]. By deducing and analyzing the quaternion differential equation, we can obtain the relationship between the quaternion obtained from the gyroscope output and the quaternion obtained from the real data.

The quaternion differential equation is expressed as follows [17]:

(3) $$\dot{q}\left(t\right)=\frac{1}{2}\omega_{nb}^{b}\cdot q\left(t\right)$$

Equation (3) is expressed in matrix form as follows:

(4) $$\left[\begin{array}{c} {\dot{p}}_{0}\\ {\dot{p}}_{1}\\ {\dot{p}}_{2}\\ {\dot{p}}_{3} \end{array}\right]=\frac{1}{2}\left[\begin{array}{cccc} 0 & -\omega_{nbx}^{n} & -\omega_{nby}^{n} & -\omega_{nbz}^{n}\\ \omega_{nbx}^{n} & 0 & \omega_{nbz}^{n} & -\omega_{nby}^{n}\\ \omega_{nby}^{n} & -\omega_{nbz}^{n} & 0 & \omega_{nbx}^{n}\\ \omega_{nbz}^{n} & \omega_{nby}^{n} & -\omega_{nbx}^{n} & 0 \end{array}\right]\left[\begin{array}{c} p_{0}\\ p_{1}\\ p_{2}\\ p_{3} \end{array}\right]\;$$

where $\omega_{nbx}^{n},\omega_{nby}^{n},\omega_{nbz}^{n}$ are the angular velocities of the relative reference frame around each axis that are acquired directly by a three-axis gyroscope. The quaternion, q, represents the process of rotation. $q=p_{0}1+p_{1}i+p_{2}j+p_{3}k$, and the imaginary unit, i,j,k, are the unit vectors of the three-dimensional space.

Using the Peano-Baker Approximation method in Equation (4) and for gyroscopes, the data output by the sensor is a discrete value; therefore, Equation (3) is expressed as follows:

(5) $$q_{k+1}=\exp(\frac{1}{2}\left[\Delta \theta_{k}\right])q_{k}$$

where Δθ is the three-axis rotation angle obtained by integrating the angular velocities of the three axes. The resulting expression is as follows:

(6) $$\left[\Delta \theta_{k}\right]=\omega_{nb}^{b}\cdot Ts=\left[\begin{array}{cccc} 0 & -\omega_{nbx}^{n} & -\omega_{nby}^{n} & -\omega_{nbz}^{n}\\ \omega_{nbx}^{n} & 0 & \omega_{nbz}^{n} & -\omega_{nby}^{n}\\ \omega_{nby}^{n} & -\omega_{nbz}^{n} & 0 & \omega_{nbx}^{n}\\ \omega_{nbz}^{n} & \omega_{nby}^{n} & -\omega_{nbx}^{n} & 0 \end{array}\right]\cdot Ts$$

where Ts is the sampling time of the gyroscope, and $\omega_{nb}^{b}$ is the output of the gyroscope in the carrier coordinate system. For the gyroscope output, $\Omega_{k}$ at *k*, we assume that $\Omega_{k}$ is obtained by superimposing a gyro bias, $\Omega_{bk}$, and a random noise, $v_{k}$, on the true value, $\Omega_{k}^{r}$. The bias of the gyroscope changes slowly over time; however, over a short time, the deviation of the gyroscope can be regarded as a constant. Thus, the output of the gyroscope is expressed as follows:

(7) $$\Omega_{k}=\Omega_{k}^{r}+\Omega_{bk}+v_{k}$$

The symbol, *r*, represents real data. Then, Equation (5) is re-written as follows:

(8) $$q_{k+1}=\exp(\frac{\left[\theta_{k}^{r}\right]}{2}+\frac{\left[\theta_{b}\right]}{2})q_{k}=\exp(\frac{Ts}{2}\cdot w_{k}^{r}+\frac{Ts}{2}\cdot (w_{bk}+v_{k}))q_{k}$$

because:

(9) $$\theta_{k}^{r}\cdot \theta_{b}-\theta_{b}\cdot \theta_{k}^{r}=Ts^{2}(w_{k}^{r}\cdot w_{bk}-w_{bk}\cdot w_{k}^{r})\;$$

For a gyroscope with a sampling rate of 100 Hz, the sampling time is $Ts=10^{-2}s$. Because the gyroscope bias is also a small value, the approximation is as follows:

(10) $$[\theta_{k}^{r}]\cdot [\theta_{bk}]-[\theta_{bk}]\cdot [\theta_{k}^{r}]\approx 0$$

Therefore, according to Equation (10), and because of the nature of the matrix function, the following is introduced:

(11) $$\exp(\frac{\left[\theta_{k}^{r}\right]}{2})\cdot \exp(\frac{\left[\theta_{bk}\right]}{2})=\exp(\frac{\left[\theta_{bk}\right]}{2})\cdot \exp(\frac{\left[\theta_{k}^{r}\right]}{2})\;$$

Then we obtain:

(12) $$q_{k+1}=\exp(\frac{\left[\theta_{k}^{r}\right]}{2})\cdot \exp(\frac{\left[\theta_{bk}\right]}{2})q_{k}=\exp(\frac{Ts}{2}\cdot w_{k}^{r})\cdot \exp[\frac{Ts}{2}\cdot (w_{bk}+v_{k})]q_{k}$$

Therefore Equation (12) is expressed as follows:

(13) $$\begin{array}{ll} q_{k+1} & =\exp(\frac{Ts}{2}\cdot w_{k}^{r})\cdot \exp[\frac{Ts}{2}\cdot (w_{bk}+v_{k})]q_{k}\\ & =\prod_{1}^{k}\left[\exp(\frac{Ts}{2}\cdot w_{i}^{r}\right)\cdot \exp(\frac{Ts}{2}\cdot v_{i})\cdot \exp(\frac{Ts}{2}\cdot w_{bi})]\cdot q_{1}\\ & =\exp(\sum_{1}^{k}\frac{Ts}{2}\cdot w_{bi}+\sum_{1}^{k}\frac{Ts}{2}\cdot v_{i})\cdot q_{k+1}^{r} \end{array}$$

Among them, $q_{k+1}$ represents the quaternary value of the current attitude angle; $q_{k+1}^{r}$ represents the quaternion corresponding to the current real attitude angle; $\exp(\sum_{1}^{k}\frac{Ts}{2}w_{bi})$ represents the deviation of the attitude angle caused by the gyro error. Equation (13) shows that the attitude angle from the gyroscope is obtained by adding an angle change caused by the deviation of the true value of the attitude angle. Therefore, the gyro bias, $w_{bi}$, is estimated by knowing the true value, $q_{k+1}^{r}$, of the attitude at any time.

### 2.3. Filter Design and Error Analysis

Because the deviation of the gyroscope changes slowly with time, when converted into the attitude angle, it is already included in the trigonometric function and becomes a nonlinear error. Using the conclusions derived above, the gyroscope bias can be expressed linearly. So we design a Kalman filter to estimate the gyroscope bias. We assume that the gyro bias is constant during a short period of time and the gyro bias is the stated quantity of our Kalman filter. The system equation is then written as follows:

(14) $$X_{k+1}=FX_{k}+Q_{k}$$

where *X* is the deviation of the three-axis gyroscope. Since the deviation is assumed to remain constant during the sampling time, *F* is set to a three-dimensional unit matrix. Q is the process noise, which consists mainly of the random noise of the gyroscope.

In Equation (13), we chose to use Newton’s iterative method to estimate $\sum_{1}^{k}\frac{Ts}{2}\cdot w_{bi}+\sum_{1}^{k}\frac{Ts}{2}\cdot v_{i}$ in each iteration. Then, from the estimated $\sum_{1}^{k-1}\frac{Ts}{2}\cdot w_{bi}+\sum_{1}^{k-1}\frac{Ts}{2}\cdot v_{i}$, by subtracting them at the last moment, we obtain the estimated gyroscope deviation, $w_{bk}+v_{k}$ at *k*, and use it as the observation, *y*, in our Kalman filter. In this way, we write our observation equation as follows:

(15) $$y=HX_{k}+R_{k}$$

*R* is the observation noise, consisting mainly of the random noise of the gyroscope, the error of the Newton iteration method algorithm, and the error of the method for acquiring the attitude angle observation. We use the long corridors in the map information to acquire the observations. Because it is much smaller than the errors of the random noise and the observation error of the attitude angle, the error in the Newton iteration method can be ignored. Therefore, we analyze only the influence of the error of the attitude angle observation method in the estimation of the gyro bias. Set the roll angle, pitch angle, and heading angle deviation caused by observation error to be Δϕ,Δθ,Δψ, respectively. Then for the actual situation:

(16) $$q_{k}=\exp(\hat{x})\cdot q_{k}^{r}(\phi +\Delta \phi ,\theta +\Delta \theta ,\psi +\Delta \psi )$$

where $\hat{x}$ is $\sum_{1}^{k}\frac{Ts}{2}\cdot w_{bi}+\sum_{1}^{k}\frac{Ts}{2}\cdot v_{i}$, i.e., the part of the gyro bias estimation when there is an angle observation noise. For the ideal case of angle observation, i.e., observation without noise:

(17) $$q_{k}=\exp(x)\cdot q_{k}^{r}(\phi ,\theta ,\psi )$$

where *x* is the calculated gyroscope bias estimation section under ideal conditions; $q_{k}$ is the quaternion solved by the gyroscope output. This yields the following:

(18) $$\exp(x)\cdot q_{k}^{r}(\phi ,\theta ,\psi )=\exp(\hat{x})\cdot q_{k}^{r}(\phi +\Delta \phi ,\theta +\Delta \theta ,\psi +\Delta \psi )$$

According to the lemma of the previous section we obtain:

(19) $$q_{k}^{r}(\phi ,\theta ,\psi )=\exp(\hat{x}-x)\cdot q_{k}^{r}(\phi +\Delta \phi ,\theta +\Delta \theta ,\psi +\Delta \psi )=\exp(\Delta x)\cdot q_{k}^{r}(\phi +\Delta \phi ,\theta +\Delta \theta ,\psi +\Delta \psi )$$

Because $q_{k}^{r}(\phi +\Delta \phi ,\theta +\Delta \theta ,\psi +\Delta \psi )$ rotates Δϕ,Δθ,Δψ on the basis of $q_{k}^{r}(\phi ,\theta ,\psi )$, the recurrence relation of quaternions is obtained:

(20) $$q_{k+1}^{r}=\left[\begin{array}{cccc} 1 & -\frac{1}{2}\Delta \theta_{x} & -\frac{1}{2}\Delta \theta_{y} & -\frac{1}{2}\Delta \theta_{z}\\ \frac{1}{2}\Delta \theta_{x} & 1 & \frac{1}{2}\Delta \theta_{z} & -\frac{1}{2}\Delta \theta_{y}\\ \frac{1}{2}\Delta \theta_{y} & -\frac{1}{2}\Delta \theta_{z} & 1 & \frac{1}{2}\Delta \theta_{x}\\ \frac{1}{2}\Delta \theta_{z} & \frac{1}{2}\Delta \theta_{y} & -\frac{1}{2}\Delta \theta_{x} & 1 \end{array}\right]\cdot q_{k}^{r}$$

The expression of the Euler angles will be different depending on the sequence in which the coordinate axis rotates around the three axes. In this paper, expression as following:

(21) $$\left\{ \begin{array}{l} \Delta \theta_{x}=\Delta \theta \\ \Delta \theta_{y}=\cos\phi \Delta \phi +\sin\phi \Delta \psi \\ \Delta \theta_{z}=\cos\theta \sin\phi \Delta \phi -\sin\phi \Delta \theta -\cos\phi \cos\theta \Delta \psi \end{array}\right.$$

The certification process for this formula is shown in Appendix A. Then:

(22) $$(e^{\Delta x}\cdot \left[\begin{array}{cccc} 1 & -\frac{1}{2}\Delta \theta_{x} & -\frac{1}{2}\Delta \theta_{y} & -\frac{1}{2}\Delta \theta_{z}\\ \frac{1}{2}\Delta \theta_{x} & 1 & \frac{1}{2}\Delta \theta_{z} & -\frac{1}{2}\Delta \theta_{y}\\ \frac{1}{2}\Delta \theta_{y} & -\frac{1}{2}\Delta \theta_{z} & 1 & \frac{1}{2}\Delta \theta_{x}\\ \frac{1}{2}\Delta \theta_{z} & \frac{1}{2}\Delta \theta_{y} & -\frac{1}{2}\Delta \theta_{x} & 1 \end{array}\right]-I)\cdot q_{k}^{r}(\theta )=0$$

At the same time, a Taylor expansion on $e^{\Delta x}$ takes its linear term to be the following:

(23) $$e^{\Delta x}\approx \left[\begin{array}{cccc} 1 & -\frac{kTs}{2}\Delta w_{x} & -\frac{kTs}{2}\Delta w_{y} & -\frac{kTs}{2}\Delta w_{z}\\ \frac{kTs}{2}\Delta w_{x} & 1 & \frac{kTs}{2}\Delta w_{z} & -\frac{kTs}{2}\Delta w_{y}\\ \frac{kTs}{2}\Delta w_{y} & -\frac{kTs}{2}\Delta w_{z} & 1 & \frac{kTs}{2}\Delta w_{x}\\ \frac{kTs}{2}\Delta w_{z} & \frac{kTs}{2}\Delta w_{y} & -\frac{kTs}{2}\Delta w_{x} & 1 \end{array}\right]$$

According to Equations (22) and (23), we obtain:

(24) $$\left\{ \begin{array}{l} \Delta w_{x}=-\frac{1}{kTs}\Delta \theta_{x}\\ \Delta w_{y}=-\frac{1}{kTs}\Delta \theta_{y}\\ \Delta w_{z}=-\frac{1}{kTs}\Delta \theta_{z} \end{array}\right.$$

### 2.4. Methods for Obtaining Observations

We choose a set of Euler angle measurements and then combine the above algorithm to estimate the gyro bias. The roll and pitch angles at this time are considered as “0”. So long as the heading angle of the foot can be obtained, we can obtain a set of Euler angles. Walking in an indoor environment, in some areas such as corridors and stair elevators, pedestrians will enter from a certain direction. The direction extracted in these specific areas can be used as a reference for the heading angle. A long straight corridor in an indoor map environment can be considered as such an area. In normal walking, pedestrians generally proceed along the direction of the corridor. Thus, long straight corridors can be used as a reference for direction correction. Assume the orientation of the corridor in the geographic coordinate system is ψ. Since there are two directions when pedestrians walk in the corridor, the observations for the heading are ψ and ψ+π. When pedestrians are walking in the corridor, we must first judge the choice of heading observations. Thus, we can use the orientation of the corridor as the real heading of a pedestrian in the corridor. The deviation of the gyroscope is estimated by solving Equation (13).

For this method of angle acquisition, we believe that only the heading angle contains errors. The roll and pitch angles are considered accurate. In this way, Equation (24) is expressed as follows:

(25) $$\left\{ \begin{array}{l} \Delta w_{x}=0\\ \Delta w_{y}=0\\ \Delta w_{z}=\frac{1}{kTs}\Delta \psi \end{array}\right.$$

Let Δψ be subject to the distribution of $N(0,\sigma^{2})$. The value of $\sigma^{2}$ is determined by the range of values of the heading angle given by the map. Then the covariance matrix of the gyro bias due to angular observation method error is as follows:

(26) $$\left[\begin{array}{ccc} 0 & 0 & 0\\ 0 & 0 & 0\\ 0 & 0 & {\left(\frac{\sigma }{kTs}\right)}^{2} \end{array}\right]$$

## 3. Experiment

**Figure 1:**
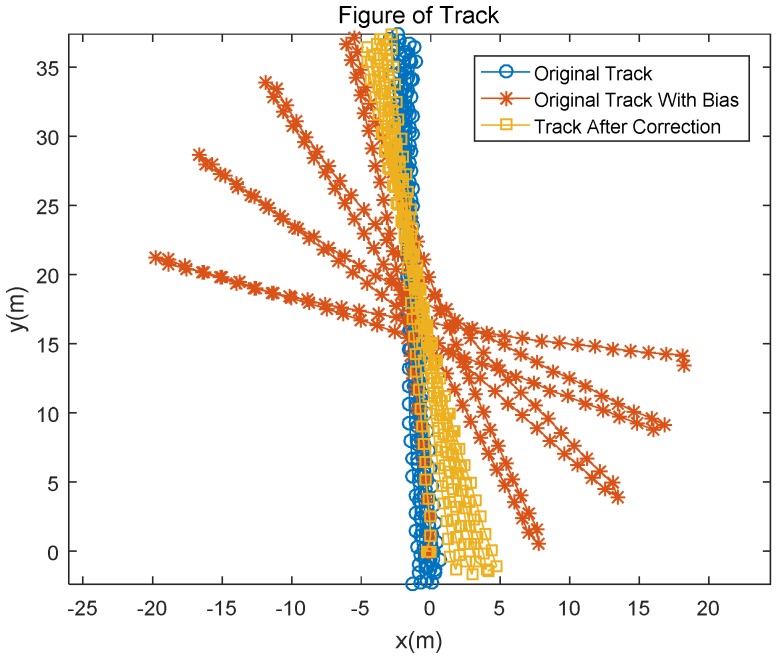
Pedestrian trajectory based on gyro bias estimation.

During the experiment, the Xsens sensor was attached to the right foot of an experimenter who walked four rounds along a corridor 39 m long and 2.4 m wide. The total duration of the experiment was 284.98 s. A preset bias was added to all the collected original gyro data of the high precision sensor. The added biases for the three axes (*x*, *y*, *z*) are [0.001, 0.001, 0.005] rad/s. In this experiment, as the foot moves, the sensor rotates and moves in three-dimensional space. Simultaneously, we observe the three-axis gyroscope at each zero-velocity update (ZUPT) point. The observation of the heading angle is the direction of the corridor, and at the ZUPT point, both the roll and pitch angles are zero. Pedestrian trajectory estimation is shown in Figure 1.

As seen from Figure 1, after adding noise, the corrected trajectory is significantly better than the track. In this experiment, due to the lack of the position of the true value of the foot, the results of the noise-added trajectory at the time of foot landing were compared with the results of the modified trajectory and the results of the Xsens high-precision sensor. The resulting parameter comparison is shown in Table 1.

**Figure 2:**
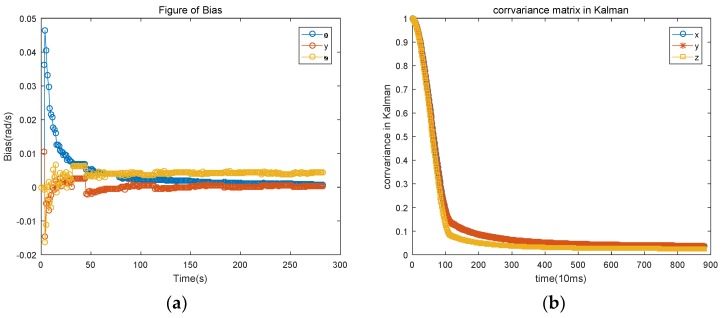
(**a**) Gyroscope bias estimation based on map information; (**b**) Variation of covariance matrix of Kalman filter with time.

According to the data in Table 1, the total error of the corrected path and the pre-correction comparison is reduced by 75% and the average error is reduced by 75%, indicating improvement of the trajectory drift caused by the deviation of the gyroscope. The gyro error estimate is shown in Figure 2.

As seen from Figure 2, there is a large fluctuation in the estimation of the gyroscope error in the initial period of time, but, with the passage of time (about 50 s in this experiment), the fluctuation gradually stabilizes to a certain value. The convergence time is not only caused mainly by the lack of the priori information of the gyroscope bias, but also by the orientation of the pedestrian, which is inconsistent with the map at the beginning of the experiment. After tracking the deviation of the gyroscope, the deviation of the gyroscope is estimated in real time. By the time a pedestrian turns, the person’s heading will change from around 0 to near −180°. Since the gyroscope cannot be in the state of ZUPT at all times, the heading change will affect the three axes at the same time. Thus, the deviation at the moment of turning (Figure 2) causes a downward jump. The gyro deviation of the convergence phase is averaged, and the three-axis deviation of the gyroscope is [0.00098, 0.00027, 0.0042], which is [98%, 27%, 84%] compared with the noise we added after the experiment. Over time, the gyroscope bias of the *Y*-axis gradually approaches the real angle and becomes more accurate.

We take the value of the bias after the deviation estimate converges to correct the trajectory with the deviation. The deviation estimate is used to calculate the trajectory comparison data obtained in the Table 2.

The comparison in Table 1 shows that, after removing the inaccuracy of the estimation due to the initial angular deviation, the overall trajectory accuracy is slightly improved. Compared with the trajectory with noise, the total error is reduced by 81%, and the average error is reduced by 81%.

**Figure 3:**
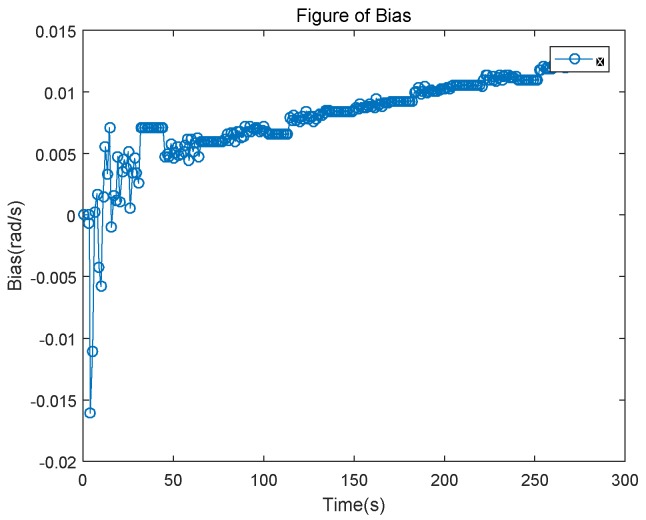
Gyroscope *z*-axis bias estimation based on map information.

For the acquired high-precision data, based on the previously added deviation, a linear bias of a slope of 5 × 10^−7^ is added to the *z*-axis of the gyroscope. Results for the gyro bias of the *z*-axis versus time are shown in Figure 3.

As shown, our estimated deviation also rises linearly after converging for 50 s. It conforms to the changing law of the bias we add. After 200 s, the data is averaged; then the estimated bias is 0.0113 rad/s. Assume the random noise average is zero; then the true bias is 0.0191 rad/s and the error is 0.0058 rad/s.

## 4. Discussion

The heading information of the long corridor of the map and ZUPT point information are used to estimate the deviation of the gyroscope. In fact, as long as it can accurately observe the angle, the algorithm can estimate the deviation of the gyroscope. The influence of angle observation error on gyroscope bias is analyzed. Regarding map information, the long corridor is just a means for obtaining the heading. Maps like escalators, long straight stairs, and even turns are used to obtain angle observations. Instead of maps, GNSS, like geomagnetism, provides angular information to estimate bias. The application is not limited to pedestrian navigation. In our experiment, based on the high-precision sensor collected by the experimenter, to correct the deviation, a fixed deviation was artificially added. In the future, the deviation of the sensor may be modeled, and the deviation of the gyroscope may be predicted.

## 5. Conclusions

To improve the accuracy of the navigation trajectory, we estimated the deviation of the gyroscope. Using the quaternion expression of the rotation process, we obtained the relationship between the rotation process calculated by the gyroscope output and the actual rotation process, which is caused by the deviation of the gyroscope. The true angle is obtained through the heading information of the long corridor of the map. According to the angle obtained by the gyroscope, the deviation of the gyroscope can be calculated. Experimental results show that the algorithm effectively estimates the bias of the gyroscope.

## Figures and Tables

**Table 1:** Comparison of gyro error estimation trajectory drift based on quaternion.

	Original Path (m)	Noise Path (m)	Corrected Path (m)
Drift Mean	0	7.10	1.75
Median drift	0	5.30	1.21
Total drift	0	1746.60	428.09
Positioning error	2.75	22.70	5.88

**Table 2:** Gyro Error Estimation Trajectory Comparison Based on Map Information.

	Original Path (m)	Noise Path (m)	Corrected Path (m)
Drift Mean	0	7.10	1.32
Median drift	0	5.30	1.00
Total drift	0	1746.60	325.34
Positioning error	2.75	22.70	4.34

## Appendix A

The expression of the Euler angles will be different depending on the sequence in which the coordinate axis rotates around the three axes. In this paper, we use the Euler angular velocity matrix has the following form [17]:

(A1) $$\left(\begin{array}{c} \dot{\phi }\\ \dot{\theta }\\ \dot{\psi } \end{array}\right)=\frac{1}{\cos\theta }\left(\begin{array}{ccc} \sin(\phi )\sin(\theta ) & \cos(\theta ) & -\cos(\phi )\sin(\theta )\\ \cos(\phi )\cos(\theta ) & 0 & \sin(\phi )\cos(\theta )\\ \sin(\phi ) & 0 & -\cos(\phi ) \end{array}\right)\left[\begin{array}{c} \omega_{x}\\ \omega_{y}\\ \omega_{z} \end{array}\right]$$

where the three-axis rotational angular velocity in the body coordinate frame, ${\left(\begin{array}{ccc} \omega_{x} & \omega_{y} & \omega_{z} \end{array}\right)}^{T}$, is the output of the gyroscope. The Euler angular velocity is ${\left(\begin{array}{ccc} \dot{\phi } & \dot{\theta } & \dot{\psi } \end{array}\right)}^{T}$. Integrating time on both sides of the equation results in the following:

(A2) $$\left(\begin{array}{c} \phi \\ \theta \\ \psi \end{array}\right)=\frac{1}{\cos\theta }\left(\begin{array}{ccc} \sin(\phi )\sin(\theta ) & \cos(\theta ) & -\cos(\phi )\sin(\theta )\\ \cos(\phi )\cos(\theta ) & 0 & \sin(\phi )\cos(\theta )\\ \sin(\phi ) & 0 & -\cos(\phi ) \end{array}\right)\left[\begin{array}{c} \theta_{x}\\ \theta_{y}\\ \theta_{z} \end{array}\right]\;$$

From the inverse matrix, we finally obtain the following:

(A3) $$\left\{ \begin{array}{l} \Delta \theta_{x}=\Delta \theta \\ \Delta \theta_{y}=\cos\phi \Delta \phi +\sin\phi \Delta \psi \\ \Delta \theta_{z}=\cos\theta \sin\phi \Delta \phi -\sin\phi \Delta \theta -\cos\phi \cos\theta \Delta \psi \end{array}\right.$$
